# Purification of infectious human herpesvirus 6A virions and association of host cell proteins

**DOI:** 10.1186/1743-422X-4-101

**Published:** 2007-10-19

**Authors:** Maria Hammarstedt, Jenny Ahlqvist, Steven Jacobson, Henrik Garoff, Anna Fogdell-Hahn

**Affiliations:** 1 Department of Biosciences and Nutrition at Novum, Karolinska Institutet, Huddinge, Sweden; 2 Department of Clinical Neuroscience, Division of Neurology, Karolinska Institutet, Stockholm, Sweden; 3 National Institute of Neurological Disorders and Stroke, National Institutes of Health, Bethesda, MD, USA

## Abstract

**Background:**

Viruses that are incorporating host cell proteins might trigger autoimmune diseases. It is therefore of interest to identify possible host proteins associated with viruses, especially for enveloped viruses that have been suggested to play a role in autoimmune diseases, like human herpesvirus 6A (HHV-6A) in multiple sclerosis (MS).

**Results:**

We have established a method for rapid and morphology preserving purification of HHV-6A virions, which in combination with parallel analyses with background control material released from mock-infected cells facilitates qualitative and quantitative investigations of the protein content of HHV-6A virions. In our iodixanol gradient purified preparation, we detected high levels of viral DNA by real-time PCR and viral proteins by metabolic labelling, silver staining and western blots. In contrast, the background level of cellular contamination was low in the purified samples as demonstrated by the silver staining and metabolic labelling analyses. Western blot analyses showed that the cellular complement protein CD46, the receptor for HHV-6A, is associated with the purified and infectious virions. Also, the cellular proteins clathrin, ezrin and Tsg101 are associated with intact HHV-6A virions.

**Conclusion:**

Cellular proteins are associated with HHV-6A virions. The relevance of the association in disease and especially in autoimmunity will be further investigated.

## Background

Human herpesvirus 6A and 6B (HHV-6A and 6B) are members of the betaherpesvirus subfamily. HHV-6B is a ubiquitous virus and causes the common childhood disease exanthem subitum [1], whereas the seroprevalence rate and pathological features for HHV-6A is unknown. Both variants are neurotropic and can cause neurological disorder [2-6] and might be potential pathologic agents in multiple sclerosis (MS), though the mechanism(s) is not understood [7]. Putatively, incorporation of host proteins into herpes virions could have implications for autoimmunity as indicated by studies of human cytomegalovirus (HCMV) [8,9]. Several reports demonstrate that host proteins are incorporated into enveloped viral particles [10-13]. However, the purity of the virus preparations and if host proteins are truly incorporated have been debated since cellular vesicles might contaminate the viral preparations during purification by sedimentation in sucrose gradients [14,15]. The drawbacks with sucrose gradients have been overcome by a switch to iodixanol gradients [16-18].

We have set up a purification protocol, including iodixanol gradient, for HHV-6A that result in carefully purified, intact and infectious viral particles. To control for cellular contaminants, the background produced by mock-infected cells was determined for every step in the purification scheme and during the characterisation of the HHV-6A virions. The cellular proteins CD46, clathrin, ezrin and Tsg101 were found to be associated with the purified HHV-6A virions. Actin was also, to a lower extent, found in the purified HHV-6A virion samples.

## Results

### HHV-6A production

**Figure 1:**
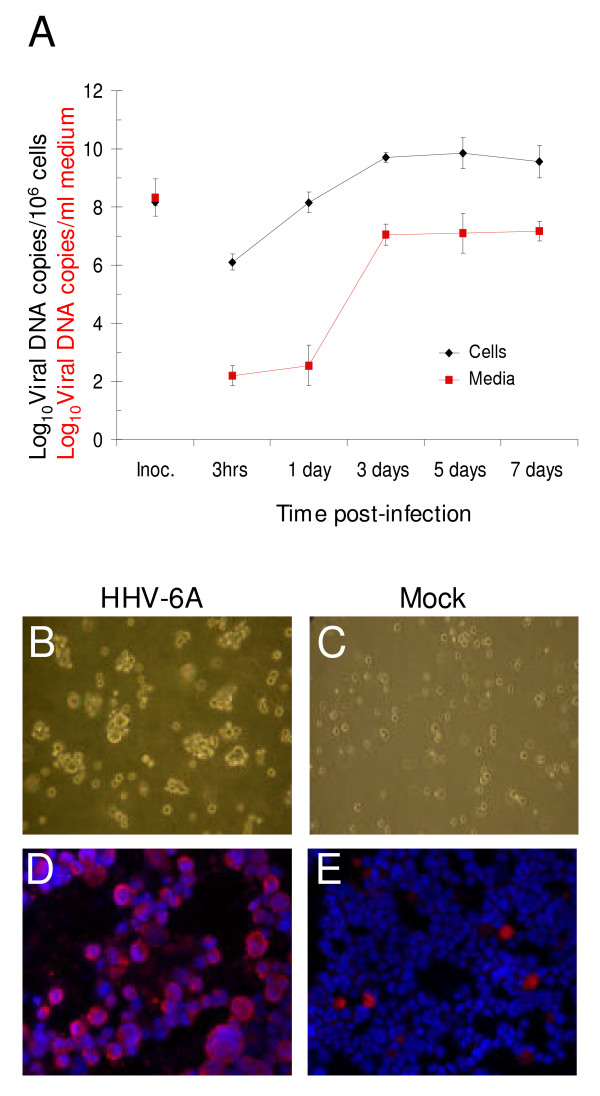
**Infections of JJHAN cells with HHV-6A (U1102)**. A. Quantifications of cellular (diamonds) and extracellular (squares) HHV-6A DNA. The log_10 _of the viral DNA copy number per ml medium or per 10^6^cells as normalized to actin, in inoculum, after 3 hpi and 1, 3, 5 and 7 dpi are shown. Results are based on three experiments and presented as mean ± standard deviation (error bars). B and C. Light microscopic analyses of HHV-6A- and mock-infected JJHAN cells at 3 dpi. Note the CPE, i.e. enlargement of cells, in HHV-6A infected cells. D and E. Detection of HHV-6 antigen gp60/110 in HHV-6A infected cells. HHV-6A-and mock-infected cells were fixed and stained for gp60/110 (red) and counterstained with DAPI (blue) to reveal cell nuclei. Light microscopy pictures are magnified 20× and fluorescent pictures 40×.

During purification and characterisation of virions, a major obstacle is contaminations of the viral preparations. The contaminations can stem from soluble proteins derived from the producer cells or from serum in the culture medium. An additional source is cellular proteins in released and co-purified cellular vesicles. To decrease the contaminations we optimised the production and collection of HHV-6A. Our first concern was the presence of high concentration of serum in the culture medium. Routinely, 10% serum was used for propagation of HHV-6A [19]. We tested whether infections could be performed with only 2% serum present. However, this led to 2 log reduction of viral DNA copies in medium as determined by real-time PCR (data not shown). We then changed from 10% serum at 24 h post infection to 2% serum and found that the viral DNA copy number in medium remained equivalent to infections grown in 10% serum. Our second concern was that release of contaminating material from the producer cells would increase with time. Therefore, the growth characteristics of HHV-6A were investigated and ideal time point for collection of viral particles as early as possible after infection was determined. Infections of JJHAN caused an increase of viral DNA copies in cells and medium over time (Fig. 1A). After 3 days, the viral DNA copy number in the medium was about 1.4 × 10^7^ per ml (= 7.1 log10) and further production gave only an insignificant increase as measured by real-time PCR analyses (Fig. 1A). This meant that at 3 days post infection (dpi), the infected cells had released 279 ± 103 viral DNA copies/infected cell (n = 3). The release of virion DNA corresponded well with the accumulation of intracellular viral DNA, which also increased rapidly to 3 dpi (Fig. 1A). Cells infected with HHV-6A displayed cytopathic effects (CPE) as enlargement of cells at day 3 (Fig. 1B), in comparison to mock-infected cells (Fig. 1C) as shown by light microscopic analyses. Altogether, we decided to perform infections in 10% serum and collect HHV-6A in 2% serum media from 1 dpi to 3 dpi. A third concern was whether the majority of cells in culture were infected with HHV-6A and thereby contributing to efficient release of virions. Immunofluorescence analysis showed that approximately 80% of the infected cells were stained by the HHV-6 specific monoclonal antibody gp60/110 at day 3 (Fig. 1D). A low number of mock-infected cells showed a diffuse red staining, but were negative for nuclear staining by DAPI and therefore most likely had non-specifically taken up rhodamine (Fig. 1E). We concluded that most cells were infected and contributed to HHV-6A production.

### Purification of HHV-6A particles

**Figure 2:**
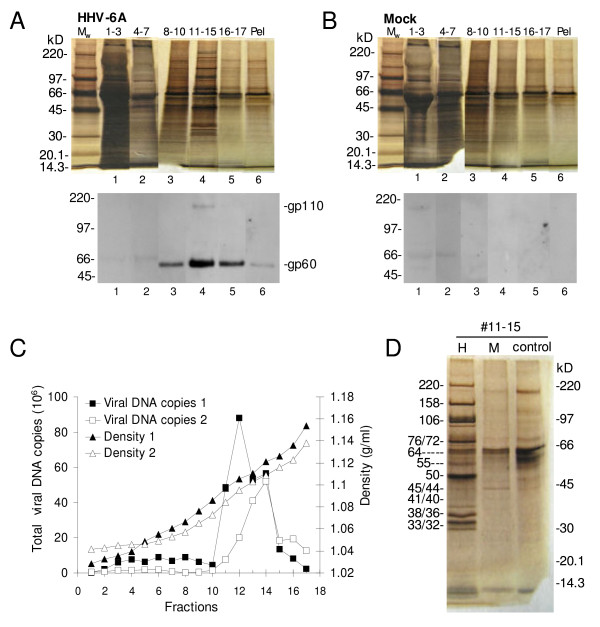
**Purification of HHV-6A**. A and B. Material in pooled iodixanol gradient fractions were concentrated by centrifugation, separated by 6–15% SDS-PAGE and visualized by silver nitrate staining or western blot using the viral specific (gp60/110) antibody. C. DNA analyses of iodixanol gradient fractions. The number of viral DNA copies in iodixanol gradient fractions of two independent experiments was measured by TaqMan based real-time PCR and the densities of the fractions by refractometer. D. The proteins in gradient purified material of HHV-6A- and mock-infected cultures were compared by SDS-PAGE stained with silver nitrate. Fresh culture medium was analyzed as a control. Estimated molecular weights in kD of the detected proteins are indicated. All samples in each separate analysis were equalized to each other based on sample volume. H, M and M_w _indicate HHV-6A, mock and molecular weight marker.

Media was collected from HHV-6A-infected cells and mock-infected control cells from 1 to 3 dpi. Importantly, the mock control was included in order to estimate the background level of contaminations during purification and analyses of HHV-6A particles. The virus and mock sample were purified in several steps as detailed in material and methods. Briefly, the collection media were clarified by short centrifugations, concentrated by ultra filtration and filtered through a 0.45 μm filter. The viral particles, and mock material, were finally purified by sedimentation on a 5–25% w/v iodixanol gradient. The iodixanol gradient fractions were concentrated into pellets by centrifugation and analyzed by SDS-PAGE followed by silver staining. As seen in lanes 1 and 2 in Fig. 2A and 2B, a considerable amount of protein containing material from both HHV-6A and mock preparations was detected in the top fractions of the iodixanol gradient while the bottom fractions contained much less proteins. A large difference in protein pattern between HHV-6A and mock samples is seen in fractions 11–15 (Fig 2A and 2B, lane 4). A number of clearly concentrated probable viral proteins are displayed in the HHV-6A sample while the mock shows a diffuse background. The presence of HHV-6A in fractions 11–15 was confirmed by parallel western blot analyses using the monoclonal HHV-6 antibody gp60/110 (Fig. 2A, lower). A strong signal for viral protein gp60 and a weak signal for gp110 were detected in these fractions. As expected, gp60/110 was not detected in the parallel mock analyses (Fig. 2B, lower). The faint bands detected in the top fractions of the mock sample gradient were most likely the result of unspecific reactions between the antibodies and serum proteins or cellular proteins (Fig 2B, lower, lanes 1 and 2). Corresponding bands were also seen in the HHV-6A blot (Fig. 2A, lower, lanes 1 and 2). Furthermore, real-time PCR analyses of viral DNA in the gradient fractions revealed a peak of HHV-6A DNA in fractions 11–15 as shown in Fig. 2C. The density of these fractions was determined to be between 1.09–1.12 g/ml. Although the majority of the initial mock material was removed during the purification procedure, a background was still present in the gradient fractions 11–15 (Fig. 2B, lane 4). We hypothesized that the background represented mostly proteins from the serum in the culture medium rather than contaminating host proteins. As a control for solely medium proteins an equal volume of fresh culture media was also concentrated, filtered and sedimented in iodixanol gradient and fractions 11–15 were analyzed in parallel with HHV-6A and mock preparations (Fig. 2D). The medium control clearly shows that the background in purified mock corresponded to culture media proteins. This background was increased if the virions were harvested in culture media containing 10% serum (data not shown). We concluded that the purification of HHV-6A virions removed a substantial amount of cellular contaminating material, but that the virions were still to some extent contaminated with soluble serum proteins.

### Purification of HHV-6A relative to cellular material

Infected cells and parallel mock cultures were metabolically labelled during a few hours with ^35^S-methionine to label synthesizing cellular and viral proteins. By this approach the purification can be followed relative to cellular material, disregarding the non-labelled serum contamination. However, the JJHAN cells were sensitive to the toxic effects of the isotope, which was manifested as an increase in background material over time. Hence, the labelling period was minimized to only four hours and thereafter virus particles were collected for four hours without additional labelling. Two time points for labelling and collection of particles after infection were chosen. The first time point was at 1 dpi when viral DNA is detected in the producer cells, but yet no viral particles are released into the media (Fig. 1A). Thus, labelled proteins present in media at 1 dpi should represent the background level of cellular proteins released from both infected and mock-infected cells. The second time point was at 3 dpi when the production level of virions was high (Fig. 1A). To enable quantitative comparisons of the amount of proteins in HHV-6A and mock preparations, the samples were equalized based on the number of living cells in the cultures at the end of collection. This is crucial as the mock-infected cells, but not HHV-6A-infected cells, divided during the collection period and hence would have given an overestimation of released cellular material.

**Figure 3:**
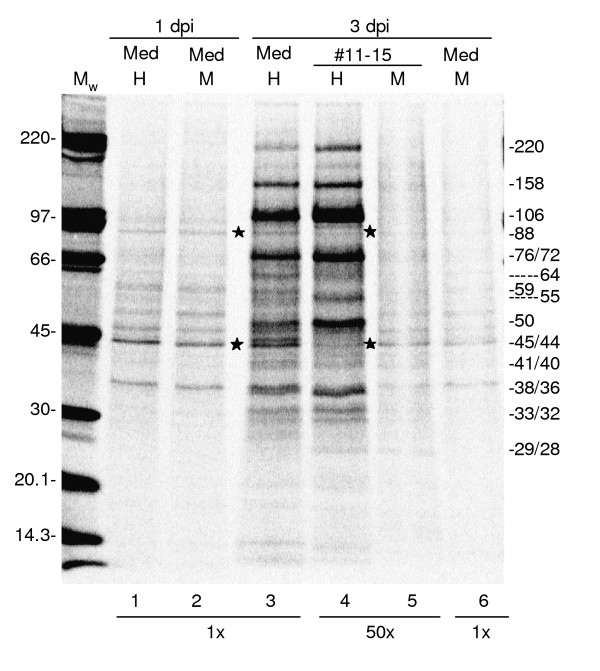
**Metabolic labelling of proteins in HHV-6A**. HHV-6A- or mock-infected cells were metabolically labelled with [^35^S]methionine between 24.5 and 28.5 hpi or 72.5 and 76.5 hpi and virions were collected without further labelling from 28.5 to 32.5 hpi or 76.5 to 80.5 hpi. HHV-6A virions and corresponding mock sample were purified, equalized based on the number of living cells in the two cultures at the end of collection period and analyzed by 6–15% SDS-PAGE. The 1 and 3 dpi media represented 2% of the total sample volume and fractions 11–15 corresponded to 98%. Estimated molecular weights in kD of the detected proteins are indicated. H, M and M_w _abbreviations as in Fig. 3. Asterisks indicate cellular proteins 88 kD and 44 kD.

In Fig. 3, the protein patterns of purified material from infected and mock cells, isolated from the iodixanol gradient peak fractions 11–15, were compared to each other and to 2% aliquots of the non-purified collection media. At 1 dpi there was no significant difference in the protein pattern between the material in non-purified collection media from HHV-6A- and mock-infected cells (Fig. 3, lane 1 and 2). All detected proteins, e.g. 44 and 88 kD, were regarded to be cellular proteins.

In contrast to at 1 dpi, there is a significant difference in the overall protein pattern between the non-purified collection media samples from HHV-6A-infected cells and the corresponding mock samples at 3 dpi (Fig. 3, lanes 3 and 6). The HHV-6A samples contain a number of strongly labelled proteins, which are not present in the mock sample. This difference is even more pronounced in the purified material in gradient fractions 11–15 (Fig. 3, lanes 4 and 5). Since these proteins had no equivalents in the 1 dpi collection media or in the 3 dpi mock samples we assumed that they likely are proteins of the viral particles. Examples are proteins of 220 kD, 158 kD, 106 kD, 76/72 kD, 50 kD and 36 kD. Notable is that the protein pattern of the metabolically labelled and purified HHV-6A virions (Fig. 3, lane 4) was very similar to the pattern found in silver stain analyses of HHV-6A virions (Fig. 2D).

Purification and recovery factors have previously been estimated by quantifications of metabolically labelled viral and cellular proteins in the starting material and final preparation [20-22]. To estimate these factors for the produced and purified HHV-6A virions at 3 dpi, the presumed viral proteins, 220, 158 and 50 kD were chosen and their amounts compared to the 44 and 88 kD cellular proteins. The measured intensities of the protein bands were adjusted according to loaded amounts on the gel, i.e. the 1 dpi and 3 dpi media samples were multiplied 50 times. For calculations of the purification factor, we first calculated ratios of the viral proteins compared to the 88 kD and 44 kD cellular proteins in 3 dpi medium and in purified particles in fractions 11–15 of the iodixanol gradient, respectively. A subsequent division of the viral to cellular protein ratio found in fractions 11–15 with the corresponding protein ratio in the 3 dpi medium, resulted in an enrichment factor between 7–15 fold (Table 1). The recovery was calculated by dividing the intensity of the individual viral proteins in fractions 11–15 with the intensity of the proteins in the 3 dpi medium. We found that the recovery of the viral proteins was about 5% (Table 1). This corresponds well with recovery rates (3.1 ± 1.5%, n = 4) calculated from real-time PCR analyses of viral DNA throughout the purification scheme.

**Table 1:** Recovery and purity of HHV-6A preparations

**Viral protein (kD)**	**Recovery (%)^1^**	**Virus to host ratio^2^**				**Purification factor^3^**	
		**3 dpi Medium**		**#1115**			
		**88 kD**	**44 kD**	**88 kD**	**44 kD**	**88 kD**	**44 kD**

220	5.4	1.8	0.4	27	4.3	15	11
158	5.2	8.3	2.0	119	19	14	9.5
50	4.1	4.2	1.0	47	7.5	11	7.5

^1^Recovery calculated as (Int_(#11–15) _/Int_(3 dpiMed × 50)_) × 100, where Int is the intensity of the proteins as quantified from metabolically labeled proteins (Fig. 4).

^2^Virus to host ratio was calculated as Int_viral protein _(#11–15)/Int_hostprotein _(#11–15) and in similar manner for 3 dpi medium.

^3^Purification factor was estimated as the viral to host ratio in #11–15 divided by the similar ratio in 3 dpi Med.

### Electron microscopy analyses

**Figure 4:**
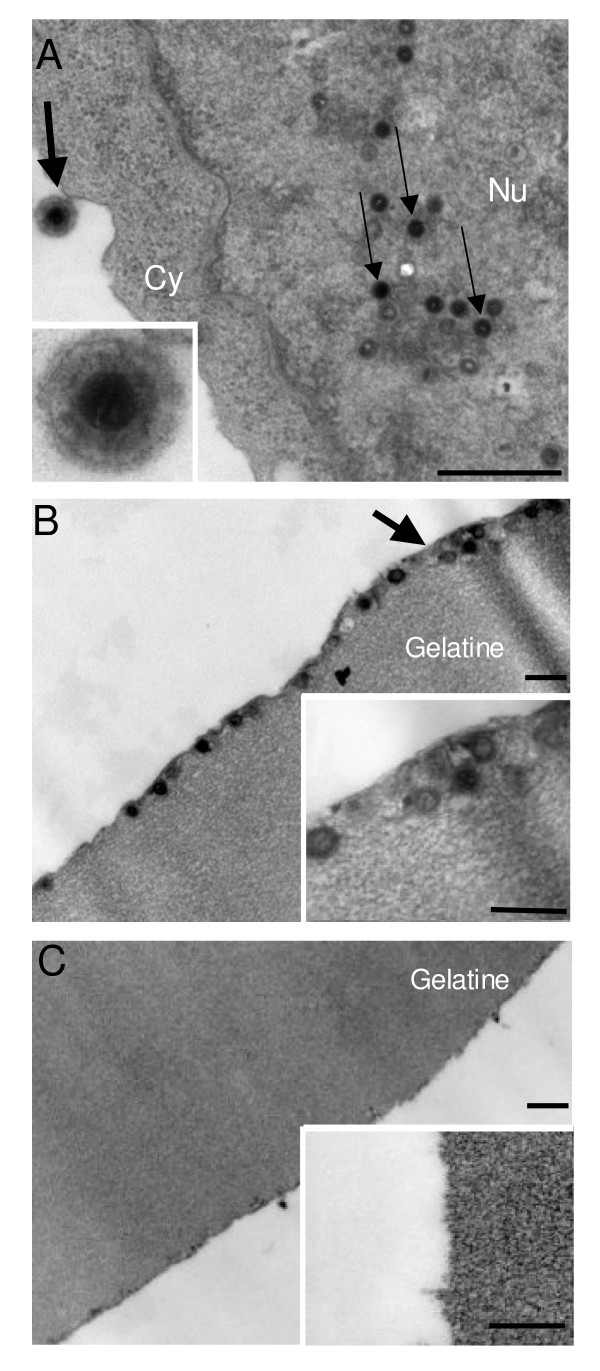
**EM thin section analyses of infected cells and of particles in iodixanol gradient fractions**. A. HHV-6A infected cells at 3 dpi. Indicated are viral nucleocapsids (thin arrows) in cell nuclei and virions released into the extracellular milieu (thick arrow and insert). The bar is 1000 nm. B and C. Material in gradient fractions 11–15. Note the intact and morphologically preserved HHV-6A particles in material from HHV-6A-infected cells (B) and their absence in samples from mock-infected cells (C). The bars are 500 nm.

To further analyze the production and purity of the HHV-6A particles, EM-analyses were performed. Shown in Fig. 4A is an HHV-6A-infected cell with a number of nucleocapsids dispersed in the nucleus (thin arrows) and a complete viral particle located extracellularly (thick arrow). About 70 of 100 counted cells contained viral particles at 3 dpi. To analyse the cellular material in iodixanol gradient fractions we pooled fractions 11–15, concentrated the samples by centrifugation, embedded the pellets in gelatine and performed EM-analyses. The analyses of the gradient peak fractions 11–15 showed apparently intact and spherical virus particles in the HHV-6A sample. Importantly, no obvious cellular material was visible in the peak fractions of HHV-6A or in the corresponding mock sample (Fig. 4B and 4C). The integrity of the purified virions was further verified by negative staining (data not shown). We conclude that purification in iodixanol gradients efficiently removes cellular contamination in form of vesicles and preserves the morphology of the virions.

### Purified HHV-6A particles are infectious

**Figure 5:**
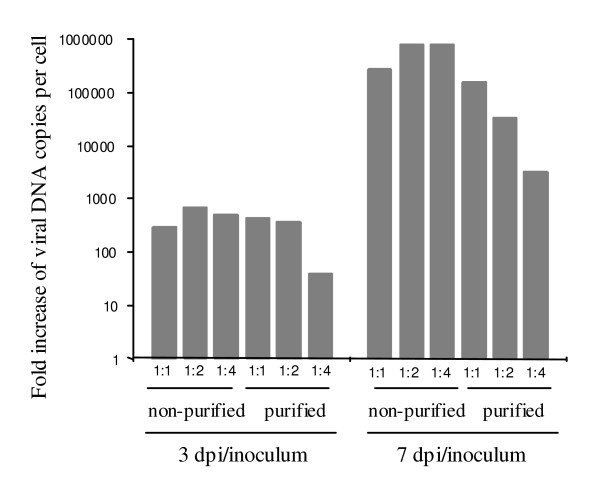
**Purified HHV-6A is infectious**. Cells were infected with non-purified collection media and purified fractions 11–15 directly or in 1:2 and 1:4 dilutions. The infectivity was measured as the fold increase of viral DNA copies per cell (normalized to actin) at 3 dpi and 7 dpi compared to the initial inocula. The fold increase was calculated by dividing the viral DNA copy number per cell with the viral DNA copy numbers in corresponding inocula.

To investigate whether the purified virions were infectious, we performed a re-infection assay (Fig. 5). Viral particles were collected and an aliquot of the non-purified 3 dpi collection medium was used as inoculum for infection of cells. The remaining collection medium went through the purification assay and the viral particles in gradient fraction 11–15 were then used as a second inoculum. Real-time PCR analysis showed that the non-purified inocula contained approximately 80 times more viral DNA copies compared to the purified inocula. Cells were infected with non-purified collection media and purified fractions 11–15 directly or in 1:2 and 1:4 dilutions. The two inocula, 1:2 and 1:4 dilutions of the inocula were used directly to infect one million cells each. The cells were lysed at 3 dpi, when the first viral particles are released from infected cells, and at 7 dpi. Viral DNA was extracted from the samples and the number of viral DNA copies per cell was determined. To compare the infection efficiency of the non-purified and purified virions, we calculated the fold increase of viral DNA copies per cell at 3 dpi and 7 dpi compared to corresponding inocula (Fig. 5).

The results show that both non-purified and purified virions gave similar fold increase in DNA viral copies/cell at 3 dpi, which suggests that both samples contained infectious particles. The same result was obtained at 7 dpi when comparing non-diluted inocula (Fig. 5). However, a lower fold increase in DNA viral copies/cell was noticed in diluted purified inoculum, especially at 7 dpi. This probably reflects the 80 times lower initial viral copy number in purified inoculum and at the 1:4 dilution the number of virions per cell might have reached a critical low point for successful infections to occur. Another consequence is that a lower amount of cells were initially infected although with same efficiency. Thus, less viral particles were released at 3 dpi that could contribute to a second round infection. When we analyzed the viral DNA copy numbers at 1 dpi, 3 dpi, 5 dpi and 7 dpi, we found that the growth curves for the non-purified inoculum and its dilutions reached a plateau after 5 dpi as in Fig. 1, which suggests that almost all cells in the culture had become infected (data not shown). In contrary, the growth curves for the purified virions and its dilutions were still increasing even at 7 dpi and probably a third round of infection would have been necessary in order to infect all cells in the culture.

In conclusion, the purified and morphological intact HHV-6A virions retained full infectivity during the purification procedure.

### Detection of cellular proteins in purified HHV-6A preparations

**Figure 6:**
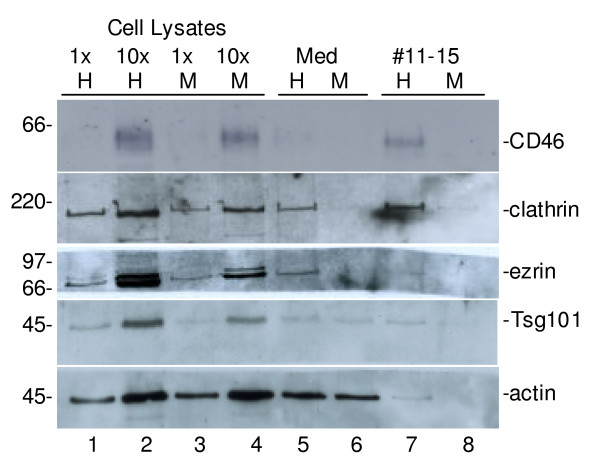
**Identification of cellular proteins associated with HHV-6A particles**. Media from HHV-6A- and mock-infected JJHAN cells were collected at 3 dpi and 2% aliquots were used directly for SDS-PAGE and western blot analyses and compared to the further purified material in iodixanol gradient fractions 11–15. The amounts of the mock (M) and HHV-6A (H) samples were equalized based on the number of living cells in the two cultures at the end of the collection. Cell lysates were analyzed in two amounts, 1× = 0.35 × 10^4 ^cells and 10× = 3.5 × 10^4 ^cells.

In an attempt to analyze the protein content of purified HHV-6A virions, with focus on cellular proteins, we performed western blot analysis on samples balanced to each other on basis of the number of living cells at the end of collection. We used a set of antibodies directed towards cellular proteins, including cytoplasmic, cytoskeletal and surface proteins. We decided to use only those antibodies that tested clearly positive in cell lysates, which excluded the antibodies directed towards for example CD4 and CD55. However anti-clathrin, -ezrin (also to some extent cross reactive to radixin and moesin), -Tsg101, -actin and -CD46 antibodies representing cellular vesicle, cytoskeletal, cytoplasmic and surface proteins gave a positive signal in cell lysates of infected and mock cells (Fig. 6, lanes 1 to 4). Next, we analyzed whether the cellular proteins were detected in aliquots of the non-purified collection media at 3 dpi. It was found that all selected proteins were to various degree detected in media collected from HHV-6A cells, but only Tsg101 and actin gave significant signals in comparable mock sample (Fig. 6, lanes 5 and 6). The latter finding indicates that at least Tsg101 and actin are present in background material released from cells, regardless if the cells were infected or not. Finally, we analyzed whether the proteins were present in gradient purified material from HHV-6A- and mock-infected cells, respectively (Fig. 6, lane 7 and 8). The results show that CD46 is clearly found with purified HHV-6A virions, but is virtually undetectable in the corresponding mock material. This suggests an association of CD46 with HHV-6A. Clathrin, ezrin and Tsg101 also appear to be concentrated to higher extent in HHV-6A particles compared to the equally purified mock sample and thus suggesting association of these cellular proteins with purified HHV-6A virions. This was confirmed by quantifications of at least two independent experiments yielding about 30 times more of CD46 in purified HHV-6A sample than in the corresponding mock sample. The numbers for clathrin was 4 times, for ezrin 13 times and for Tsg101 4 times. Actin was also detected in the purified HHV-6A virions but only 2 times more when compared to the corresponding mock sample. However, due to low level of signal to noise ratios, the quantifications were only approximate.

## Discussion

The goal of this study was to establish a purification method enabling protein analyses, with focus on associated cellular proteins, of highly purified, morphology preserved and still infectious HHV-6A virions. For these analyses it is important that the isolated particles are as free as possible from cellular contaminations. To this end, we modified a purification method previously shown to yield highly purified retroviral particles [17,18]. First, to avoid extensive contaminations, we collected the HHV-6A particles during a short time interval soon after the infection. Second, the collected virus particles were sedimented in iso-osmotic iodixanol gradients which efficiently separate soluble proteins, cellular vesicles and viral particles from each other in contrast to sucrose gradients, which can lead to viral preparations contaminated by cellular vesicles [14-16]. Another disadvantage with sucrose gradients is demonstrated by extensive aggregation of HCMV particles during sedimentation, which may influence the infectivity of the purified virus [23,24]. Third and most importantly, controls for release of cellular material and contamination of purified preparations were included. For this purpose we analyzed material released from comparable mock-infected cells and fresh culture media.

The efficiency of purification was followed by thin section EM analyses to investigate the overall content of the viral preparations [22]. The result showed that the viral preparations were purified from contaminations in form of large aggregates or cellular vesicles. Besides, the viral particles had intact morphology since no stain penetrated the particles in negative stain analyses. Despite efficient purification, the virions were to some extent contaminated by serum proteins as seen in sensitive silver stain analyses [25]. We made an effort to further reduce the level of serum contamination by slowly reducing the level of serum in cell culture to only 0.2%. However, the production of virus particles was decreased and the method was therefore abandoned. It should be noted, that HCMV [26], Epstein-Barr virus (EBV) [27] and Human herpesvirus 8 (HHV-8) [28] can be purified to levels where serum bands are not detected by the 100 times less sensitive Coomassie Brilliant blue staining [25]. However, comparable control samples have seldom been shown, which makes the purity difficult to estimate. Purifications of HHV-6B have often included time consuming sedimentations in cesium chloride gradients [29,30]. Notable is also that purification protocols for other viruses have given higher purification folds and recovery rates than those we obtained. We have, on the other hand, aimed to reduce the contamination already by short collection times at a suitable time point. Previous purification protocols have often not assessed the infectivity capacity of the final product. We demonstrate that HHV-6A particles purified in iodixanol gradients are infectious. Our assay might be an alternative method, if fast and mild one-day purification of viable viral particles is required.

The HHV-6A viral preparations were to low extent contaminated by cellular proteins as seen in metabolic labelling experiments. However, the cells were sensitive to the toxic effects of the isotope, which was manifested in increased background with prolonged labelling times. Hence, the protein background found in purified preparations during metabolic labelling might not be fully representative of the protein contamination level of non-labelled HHV-6A preparations. It should be noted that the background level of metabolically labelled material in these analyses could be influenced by three parameters. First, increase of cell number in the mock culture compared to the HHV-6A-infected culture may result in overestimating of released cellular material from mock culture. Therefore, the analyses were based on the number of living cells in the cultures at the end of collection of virus particles. Second, cells are dying during the experiment and material is released into the culture media. The cells were counted throughout the experiments and the number of dead cells in HHV-6A- and mock-infected cultures did not differ extensively (data not shown). Also, that the cells were washed at every step of the experiment including at the start of labelling and before collection of particles, which reduce the amount of released soluble material in the collection media. Third, HHV-6A-infected cells might react differently from mock-infected cells and due to the infection release a higher extent of cellular material or a different set of proteins into the collection media, which may result in an increase of cellular proteins in purified virions. However, two representative cellular proteins, 44 kD and 88 kD, are found at similar levels in the purified samples of both HHV-6A and mock at both 1 dpi (data not shown) and 3 dpi, indicating that our control consisting of material released from mock-infected cells is comparable to the proportion of material released from HHV-6A-infected cells, Therefore, we conclude that the cellular proteins CD46, clathrin heavy chain, ezrin, and Tsg101 are associated with the purified HHV-6A virions. Actin might also be associated with purified HHV-6A since it was found at a level of 2 times more than in the corresponding mock sample. However, it is doubtful if 2 times more is significant and therefore we just conclude that actin was present in the purified HHV-6A sample. CD46 is the receptor for HHV-6A [31] and as such it can be discussed whether soluble or vesicle bound CD46 released from the infected cells might bind to the produced HHV-6A virions and account for the high association of CD46 with purified HHV-6A virions. However, the issue of unspecific attachment of released proteins to produced virions has been examined before and found to not significantly contribute to the number of associated proteins [17]. Association of CD46 with HHV-6A viral particles has previously been indirectly shown in MS patient samples. In that study, HHV-6A particles from 4 out of 42 MS patient sera were isolated using an immunoaffinity column comprised of immobilized monoclonal antibody towards CD46 [32]. Our present results confirm a direct association of CD46 with HHV-6A virions.

Incorporation of host proteins, like complement proteins, into viral particles may exert beneficial effects for the virus as protection from complement mediated lysis [13,33,34]. However, incorporation of host material may also result in detrimental immune responses, such as autoreactive B- and T cells [9,35]. For instance, addition of myelin basic protein to the enveloped virus VV was shown to be important for autoimmunity and induction of encephalomyelitis [36]. Given that HHV-6A forms a latent infection in the brain [37] and that reactivation of the virus has been detected in oligodendrocytes in MS patients [7], it is of high relevance to investigate the overall protein content of any HHV-6A particles and especially in those released from human oligodendrocytes and to analyze the subsequent immunological events. However, such a study is impeded due to difficulties in obtaining and propagating sufficient amount of human oligodendrocytes. Our present study is a first attempt to investigate these issues and the results show that a number of diverse cellular proteins are associated with purified HHV-6A particles produced in JJHAN cells. This opens up for the possible incorporation of other cellular proteins, such as myelin in HHV-6A particles produced in oligodendrocytes, and further investigations of mechanisms for induction of autoimmune reactions.

## Conclusion

HHV-6A virions were purified using iodixanol gradient, which efficiently separate cellular vesicles and virions. The purification yielded morphology intact and infectious particles. Purity was assessed in each step of the purification procedure by comparing with a control consisting of material released from mock-infected cells. CD46, clathrin, ezrin and Tsg101 were found to be several times more concentrated in the purified virus sample than in the similarly purified sample from mock infected cells. This suggests that these cellular proteins are specifically associated with the virions.  

## Methods

### Viruses and cell lines

HHV-6A (U1102) was propagated in the Human T-cell lymphoblastoid cell line JJHAN as previously described [38].

### HHV-6A infection

JJHAN cells were washed with phosphate buffered saline (PBS) and infected with clarified inocula containing about 1.3 × 10^8 ^DNA viral copies of HHV-6A (U1102) per 10^6 ^cells. After 3 h incubation, cells were washed and maintained in RPMI containing 10% FCS for 24 h. The cells were washed and RPMI containing 2% FCS was added and incubation continued. At time points 3 h, 1, 3, 5 and 7 days post infection (dpi), cells and media were harvested for DNA extraction. Samples for immunofluorescence assay and electron microscopy were taken at 3 dpi. Mock infection was carried out using clarified culture media of uninfected JJHAN cells.

### Production, isolation and purification of viral particles

HHV-6 particles and mock material were collected in RPMI containing 2% FCS media at chosen time intervals, mostly 1 dpi to 3 dpi. Media was clarified by centrifugations twice for 10 min at 2000 × *g *in a Heraeus Labofuge 400R centrifuge and once for 20 min at 39 813 × *g *and 10°C in a Beckman JA17 rotor and then concentrated by ultra filtration in Millipore Amicon Ultra-15 tubes (Millipore Corporation, Bedford MA, USA) at 3939 × *g *for repeated 10 min intervals at 20°C in a Heraeus Labofuge 400R centrifuge until about 1 ml remained. The concentrated media were filtered through a Millipore low protein binding Durapore 0.45 μm filter (Millipore Corporation, Bedford MA, USA), layered on top of a 5 to 25% (w/v) iodixanol gradient (Axis-Shield PoC AS, Oslo, Norway) and particles were purified by sedimentation for 1.5 h at 160 000 × *g *at 4°C in a Beckman SW41 rotor. The gradients were fractionated from the top (700 μl/fraction) and virus containing fractions were detected by real time PCR, pooled, diluted by TNE (50 mM Tris-HCl pH 7.4, 100 mM NaCl, 0.5 mM EDTA) and concentrated by centrifugation in a Beckman SW41 rotor at 151 260 × *g *at 4°C for 1.5 h. Alternatively, individual gradient fractions were diluted in TNE and concentrated by centrifugation at 34 000 × at 10°C for 1.5 h in a Beckman JA18.1 rotor.

The refractive index (R_i_) of the gradient fractions were measured and their densities (δ) calculated by the formula δ = 3.362 × R_i_-3.483.

Cells were lysed in 1% Nonidet P-40, 50 mM Tris-HCl pH 7.6, 150 mM NaCl, 2 mM EDTA, 1 μg/ml phenyl methyl sulfonyl fluoride on ice and the lysate was clarified by a 5 min 6000 rpm (3709 × *g*) centrifugation in a table top Eppendorf centrifuge.

### DNA extraction and quantitative real-time TaqMan PCR

Extraction of DNA and determination of viral DNA copies for HHV-6A was performed as previously described [38]. The viral DNA copy number (N_HHV-6A_) per one million cells was calculated by the following formula: (N_HHV-6A_) × (N_β-actin_^-1^) × 0.5 × 10^6^. Final number of viral DNA copies in collected culture media was expressed as viral DNA copies/ml.

### Analysis by SDS-PAGE and silver staining

Samples were separated on SDS 6–15% gradient polyacrylamide gel electrophoresis (PAGE) as described [17]. The samples were normalized to each other by volumes of the samples or by the number of living cells from which the samples were produced. Silver stain was performed essentially as described [39]. The gel was fixed in 40% (v/v) ethanol and 10% (v/v) acetic acid for 1 h, washed with water for 15 min, incubated twice for 30 min with 0.05% (w/v) 2-,7-napfthalenedisulfonic acid disodium salt, washed with water four times for 15 min and incubated for 30 min in a 0.8% (w/v) AgNO_3_, 0.34% (v/v) NH_3 _and 18 mM NaOH mixture. The gel was washed with water, developed with 0.01% (w/v) citric acid, 0.01% (v/v) formaldehyde mixture and the reaction was stopped with 5% (v/v) acetic acid. The gel was washed with water, dried and scanned by a CanoScan 8400F (Canon Svenska AB, Solna, Sweden).

### Western blot analyses

The primary antibodies used were anti-gp60/110 (MAB8537) and anti-actin (MAB1501-R) (Chemicon International, Temecula, CA, USA), the anti-Tsg101 (sc-6037), anti-ezrin (sc-6407), anti-clathrin HC (sc-6579) and anti-CD46 (sc-9098) (Santa Cruz Biotechnology, Inc., Santa Cruz, CA, USA). The secondary antibodies used were horseradish peroxidase conjugated donkey anti-rabbit IgG (NA934), sheep anti-mouse IgG (NA931) (Amersham Pharmacia Biotech, Uppsala, Sweden) and donkey anti-goat IgG (sc-2020, Santa Cruz Biotechnology). Western blot were performed as described [17]. Quantifications of detected proteins were performed by using a Versa Doc Imaging system, model 4000 and the QuantityOne program from Bio-Rad Laboratories (Hercules, CA, USA).

### Indirect Immunofluorescent Assay

Fluorescence microscopy analysis for gp60/110 was conducted as previously described [38].

### Transmission Electron Microscopy

Negative staining of virions in gradient fractions and preparation of JJHAN cells was done as described [38,40]. Material in iodixanol gradient fractions 11–15 were concentrated by centrifugation and the pellets were embedded in a droplet of warm 10% gelatine in PBS (37°C) for 10 min. The samples were fixed, postfixed, sectioned and stained [38]. Sections were examined in a Tecnai 10 (Fei Company, Eindhoven, The Netherlands) microscope operated at 80 kV equipped with a MegaView 3 digital camera. The images were acquired and analyzed with the image processing software Analysis (Soft Imaging system GmbH, Munster, Germany).

### Metabolic labelling

The cells were infected for 3 h and maintained in RPMI containing 5% FCS for 21 or 69 h. The cells were washed with PBS and incubated for 30 min in low-methionine DMEM medium (3.0 μg of methionine/ml) (medium no. 991303; National Veterinary Institute, Uppsala, Sweden) supplemented with phosphate up to the regular concentration of 125 μg/ml, 2 mM glutamine, 5% FCS, 20 mM HEPES, 100 U of penicillin/ml and 100 μg of streptomycin/ml. The cells were labelled for 4 h in fresh similar media supplemented with 100 μCi/ml of [^35^S]methionine. The cells were washed with PBS, RPMI containing 2% FCS supplemented with an excess of unlabeled methionine (300 μg/ml) was added and virus collected from 28.5 to 32.5 or 76.5 to 80.5 hpi. The particles were collected, purified and analysed by SDS-PAGE. The gel was fixed in 10% trichloroacetic acid-40% methanol for 30 min at RT before being dried and exposed to a BAS-MS2025 image plate from Fujifilm (Science Imaging Scandinavia, Nacka, Sweden). The amount of radioactivity in proteins was measured using a Molecular Imager FX, and the QuantityOne program from Bio-Rad Laboratories (Hercules, CA, USA).

## Competing interests

The author(s) declare that they have no competing interests.

## Authors' contributions

MH and JA carried out the purification and analysis of the study with equal contribution and drafted the manuscript. AF-H, SJ and HG participated in its design and coordination and helped to draft the manuscript. All authors read and approved the final manuscript.
